# Associations of Dietary Intakes of Calcium, Magnesium, and Soy Isoflavones With Bone Fracture Risk in Men: A Prospective Study

**DOI:** 10.1002/jbm4.10563

**Published:** 2021-11-23

**Authors:** Yong Cui, Hui Cai, Wei Zheng, Xiao‐Ou Shu

**Affiliations:** ^1^ Division of Epidemiology, Department of Medicine, Vanderbilt‐Ingram Cancer Center, Vanderbilt Epidemiology Center Vanderbilt University Medical Center Nashville TN USA

**Keywords:** POPULATION‐BASED PROSPECTIVE STUDY, OSTEOPOROSIS, FRACTURE RISK AND PREVENTION, INTAKES OF CALCIUM AND MAGNESIUM, SOY ISOFLAVONES

## Abstract

The role of dietary factors in osteoporotic fractures in men is underinvestigated. We examined the associations of dietary intakes of calcium, magnesium, and soy isoflavones with risk of osteoporotic fractures in the Shanghai Men's Health Study. Included in this prospective study were 61,025 men aged 40 to 74 years at study enrollment (2002–2006). The cohort was followed up via in‐person surveys for occurrence of bone fractures, major diseases, and survival status. Multivariable Cox regression was applied to evaluate the associations of variables under study (ie, dietary intakes of calcium, magnesium, and soy isoflavones) with incidence of osteoporotic and non‐osteoporotic fractures, measured by hazard ratio (HR) and 95% confidence interval (CI). During a median follow‐up of 9.5 years, 1.2% and 3.4% of participants experienced osteoporotic or non‐osteoporotic fractures, respectively. Dietary calcium intake was inversely associated with risk of osteoporotic fractures with adjusted HRs of 0.78 (95% CI 0.60–1.02) and 0.27 (95% CI 0.13–0.56), respectively, for intake levels of 401 mg/d and >1000 mg/d versus ≤400 mg/d. Higher magnesium intake was associated with increased risk of osteoporotic fractures after adjusting for dietary calcium intake, with HRs of 1.27 (95% CI 0.97–1.66) and 2.21 (95% CI 1.08–4.50), respectively, for intakes of 251 mg/d and >450 mg/d versus intake ≤250 mg/d. High soy isoflavone intake was associated with a 25% reduction of osteoporotic fracture risk (HR = 0.73, 95% CI 0.56–0.97 for soy isoflavone intake >45.2 mg/d versus <21.7 mg/d). Dietary intakes of calcium, magnesium, or soy isoflavones were unrelated to the risk of non‐osteoporotic fractures. Our study added to the evidence that dietary calcium intake was inversely associated with a reduced risk of osteoporotic fractures in a dose–response fashion, while high magnesium intake was associated with an increased risk. Our study also revealed a novel association between higher soy isoflavone consumption and osteoporotic fractures in men. © 2021 The Authors. *JBMR Plus* published by Wiley Periodicals LLC on behalf of American Society for Bone and Mineral Research.

## 1 Introduction

Osteoporosis is a progressive systemic skeletal disease characterized by low bone mass and microarchitectural deterioration of bone tissue, with a consequent increase in bone fragility and susceptibility to fractures.^(^ ^1^, ^2^ ^)^ Osteoporotic fractures are associated with increased disability, morbidity, and mortality, especially among the elderly population, and impose a considerable economic burden on health services.^(^ ^2^, ^3^ ^)^

Studies have shown that several nutritional and dietary factors may influence the risk of osteoporosis and osteoporotic fractures.^(^ ^4^, ^5^, ^6^, ^7^ ^)^ The vast majority of previous studies, however, were conducted in women. Evidence‐based information on the roles of these modifiable factors in osteoporotic fractures in men is limited and much needed for the development of non‐pharmacologic preventive strategies.

Calcium is a well‐established nutritional factor required to maintain bone homeostasis and plays an important role in bone development. Low calcium intake has been linked to lower bone mineral density and increased risk of osteoporosis and bone fractures.^(^ ^8^ ^)^ Calcium intake has been recommended for osteoporosis prevention; however, the optimal level of calcium intake for the prevention of osteoporosis has been much debated,^(^ ^9^, ^10^, ^11^ ^)^ and the recommended amount of calcium intake varies largely by country. For instance, daily calcium recommendations for individuals older than 50 years are 500 mg by the World Health Organization, 700 mg in the UK, 800 mg in Scandinavia, 1000 mg (men) and 1200 mg (women) in the United States, and 1300 mg in Australia and New Zealand.^(^ ^10^, ^11^, ^12^ ^)^ Furthermore, direct evidence to support these recommendations for osteoporotic fracture prevention is lacking.

Magnesium is another essential micronutrient and a major component of bone.^(^ ^13^ ^)^ Although magnesium deficiency has been shown to be deleterious to skeletal health, studies have reported that high concentrations of magnesium have an inhibitory effect on osteoblast differentiation and mineralizing activity, and high levels of dietary magnesium intake may potentially pose fracture risks.^(^ ^13^, ^14^, ^15^, ^16^ ^)^ Furthermore, studies have also shown that magnesium may interact with calcium and/or vitamin D, interfere with calciotropic hormones, and has been known as a natural calcium antagonist.^(^ ^7^, ^13^, ^17^, ^18^ ^)^ Thus, it is feasible that dietary magnesium intake may also influence the risk of osteoporosis‐related fractures. To date, information on the relationship between dietary magnesium intake and osteoporotic fracture risk is scant.

Isoflavones, rich in soybean and soy‐based products, are major types of phytoestrogen, with a noticeable property as a natural selective estrogen receptor modulator.^(^ ^19^ ^)^ In vitro experiments and in vivo animal studies have shown that isoflavones have potential bone‐specific effects via estrogenic/antiestrogenic effects and other biologic mechanisms.^(^ ^6^ ^)^ Epidemiological and clinical evidence support that dietary isoflavones attenuate menopause‐induced osteoporotic bone loss and fractures among women.^(^ ^20^, ^21^, ^22^, ^23^, ^24^, ^25^ ^)^ However, the association of isoflavone intake with osteoporotic fractures in men remains largely unknown.

In the present study, we investigated the incidence of osteoporotic fractures and evaluated its associations with dietary calcium, magnesium, and soy isoflavone intakes in a prospective observational cohort of more than 61,000 adult men.

## 2 Materials and Methods

### 2.1 Study population

Participants of the study were drawn from the Shanghai Men's Health Study (SMHS), a large population‐based prospective cohort study conducted in urban Shanghai, China. Detailed descriptions of the study design and methods have been published elsewhere.^(^ ^26^ ^)^ Briefly, 61,469 men aged 40 to 74 years without a cancer history were recruited from eight typical urban communities in Shanghai between 2002 and 2006, with a 74.0% participation rate. At the study enrollment, each participant signed a consent form and completed an in‐person survey conducted by trained interviewers. The baseline information collected included sociodemographic characteristics, dietary habits including soy intake, physical activity, and other lifestyle factors, as well as medical history. Anthropometric measurements were also taken. The cohort has been followed up, starting from enrollment and ending between 2012 and 2017, through a combination of three in‐person surveys (at years 2004–2008 for first, 2008–2012 for second, and 2012–2017 for third follow‐ups) to update exposure information and collect information on changes of health status, including bone fractures and vital status. Annual record linkages with the vital statistics registry were carried out to ensure a complete ascertainment of mortality information. Response rates for these three in‐person follow‐up surveys were 97.6%, 91.9%, and 93.0%, respectively. The SMHS was approved by the institutional review boards of all participating institutions.

### 2.2 Study variables and covariates assessment

Dietary information was collected using a validated food‐frequency questionnaire (FFQ) at baseline and at the first follow‐up survey.^(^ ^27^ ^)^ A total of 81 food items were included in the SMHS FFQ. For each food item or food group, subjects were asked how frequently (daily, weekly, monthly, yearly, or never) they consumed the food or food group, which was followed by a question on the amount consumed in lians per unit of time. Lian is a unit of weight in China (1 lian = 50 g). Soy intake assessed in the study included consumption of tofu, soy milk, fresh soybeans, and other soy products by both frequency and amount of intake. Daily intakes of calories, macro‐ and micronutrients, calcium, magnesium, protein, vitamin D, soy isoflavones, and major isoflavone components (genistein, daidzein, and glycitein) were derived from FFQ data by summing the products of individual food intake amounts and nutrient contents of food items based on the Chinese Food Composition Tables.^(^ ^27^, ^28^ ^)^ To improve the dietary assessment, we averaged baseline and first follow‐up dietary intake data (FFQ) and applied them in the current study. For those who did not complete the dietary intake information at the first follow‐up (17%), only baseline data were used.

### 2.3 Outcomes

The primary outcome of interest was occurrence of osteoporotic fractures, which was primarily based on self‐reported information collected during the follow‐up surveys. During in‐person follow‐up surveys, participants were asked if they had a bone fracture since the last survey. If a participant answered “yes,” he was asked to provide further information on time (month and year), anatomic site(s), and cause of fracture. Fracture sites were coded with ICD‐9 (for first and second follow‐ups) and ICD‐10 (for third follow‐up). Anatomic sites commonly associated with osteoporotic fractures include ICD‐9 codes 805, 806, 807, 808, 810, 812, 813, 818, 819, 820, 821, 822, 823, 824, and ICD‐10 codes S22, S32, S42, S52, S72, S82, and M80. For causes of fractures, participants could select (i) car accident or physical trauma, (ii) fall when riding a bicycle, (iii) fall by sliding/fall from standing height, (iv) fall down from a high place (providing height in meters), and (v) others (specify the cause). Osteoporotic fractures were defined as low‐trauma bone fractures (eg, due to falls by sliding/from standing height) and occurring in anatomic sites commonly associated with osteoporosis.^(^ ^24^, ^29^ ^)^ Fractures with a Warriner's score ≥7 were considered as most likely due to osteoporosis, whereas other fractures with the lowest attribution scores (1 to 3) or mid‐range scores (4 to 6) were not considered as osteoporotic fractures.^(^ ^29^ ^)^ Non‐osteoporotic fractures included any fractures other than osteoporotic fractures. Because trauma, which is unrelated to diet, is the major cause of non‐osteoporotic fractures, we also included non‐osteoporotic fractures as a comparison group to evaluate the validity of outcome assessment and study findings.^(^ ^30^, ^31^ ^)^ Pathological fracture due to neoplasms was not included as study outcome and was handled by censoring (see below).

### 2.4 Statistical analysis

**Fig. 1:**
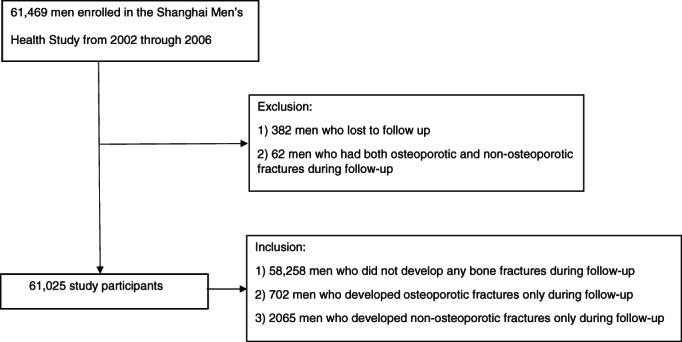
Flat Chart for Participant Selection Process.

Among 61,469 SMHS participants, we excluded 382 men who were lost to follow‐up and 62 men who had both non‐osteoporotic and osteoporotic fractures, leaving a total of 61,025 participants for the current study (Fig. 1). Chi‐square tests and Bonferroni method for *p* adjustment for categorical variables and Bootstrap adjustment for mean comparisons for continuous variables were performed to compare baseline characteristics of participants who developed fractures and those who remained event‐free. Multivariable Cox regression model was applied to evaluate the associations of variables under study (ie, dietary calcium intake, dietary magnesium intake, and soy isoflavone intake) with incidence of osteoporotic and non‐osteoporotic fractures, measured by hazard ratio (HR) and 95% confidence interval (CI). Entry time was the date of participant enrollment, and exit time was date of fracture occurrence, date of cancer, stroke, or myocardial infarction diagnosis (due to a concern that these events and their associated treatments may change a participant's dietary habits), or date at death or last follow‐up, whichever came first. Dietary calcium, magnesium, and isoflavone intakes were analyzed as both continuous and categorical variables. Because the recommended amount of calcium intake varies by countries and organizations, ranging among 500, 700, 800, 1000, and 1300 mg/d,^(^ ^10^, ^11^, ^12^ ^)^ we categorized calcium intake into six categories from ≤400 (the lowest quintile in our study population = 418 mg/d), 401–500, 501–600, 601–800, 801–1000 mg/d to >1000 mg/d to facilitate a comparison of our findings to various recommendations. We categorized magnesium intake into six categories, starting at 250 mg/d (the lowest quintile = 256 mg/d), followed by 50 mg increments from ≤250 to >450 mg/d. Soy isoflavone intake was categorized by quartile distributions. Covariates adjusted for in the models included known/suspected risk factors for bone fractures based on literature and factors that were significantly associated with bone fracture risk in the univariate analysis of our own data. Proportional hazard assumption was assessed by adding an interaction term between an exposure and time (exposure*log [time]) in the model for testing. No violation of the proportionality assumption was discovered. Two models were used for the primary analysis. In model 1, covariates included age (continuous variable), educational level (less than high school/high school graduate/higher than high school), cigarette smoking status (never/ever), alcohol consumption (yes/no), regular exercise (yes/no), body mass index (BMI, continuous variable), calcium supplement use (non‐user/ever‐user), comorbidity (Charlson's score, 0/1/≥2), fracture history (yes/no), and dietary intakes of calories, protein, fat, and vitamin D (quartiles). In model 2, dietary calcium and magnesium intakes were mutually adjusted for in addition to the covariates included in model 1. Because of a low proportion of missing values in our data (missing rates from 0.04% to 1.32%), we assigned missing values with the most frequent category for discrete variables or a median for continuous variables. We examined shapes of dose–response associations using restricted cubic spline regression with three knots, for which the positions were automatically selected.^(^ ^32^ ^)^ Wald statistics was used for testing linear and non‐linear relationships. We also conducted two sensitivity analyses. In the first one, to examine the potential influence of calcium supplement use on our study results, we excluded 4856 men from the study (approximately 8.0% of all participants) who were calcium supplement users. In the second sensitivity analysis, we included 62 men who had both osteoporotic and non‐osteoporotic fractures and had been excluded from the primary analysis to evaluate if the exclusion had affected the study results. Among them, 29 men who experienced osteoporotic fractures first were categorized as osteoporotic fractures, and 33 men who had non‐osteoporotic fractures first were categorized as non‐osteoporotic fractures. All statistical tests were based on two‐tailed probability and a significance level set at alpha (α) <0.05.

## 3 Results

During a median follow‐up of 9.5 years, 702 SMHS participants developed osteoporotic fractures (1.2%), 2065 developed non‐osteoporotic fractures (3.4%), and 58,258 remained free of any bone fractures. Table 1 shows participant characteristics by no bone fractures, osteoporotic fractures, or non‐osteoporotic fractures. Compared with those without any bone fractures during follow‐up, participants who experienced osteoporotic fractures were more likely to be older, especially older than 60 years, do exercise regularly, have lower BMI, have a comorbidity or a history of fracture, and have lower daily intakes of calories, protein, calcium, magnesium, and vitamin D. No statistically significant differences were noted between these two groups on income, education, smoking status, alcohol consumption, consumption of multivitamins, vitamin D or calcium supplementation, and fat intake. In contrast, participants who experienced non‐osteoporotic fractures during follow‐up, compared with those without any bone fractures, were more likely to be younger, ever‐smokers or ever‐drinkers, not do exercise regularly, take calcium supplements, have a comorbidity or a history of fracture, and have higher daily intakes of calories, protein, fat, calcium, and magnesium.

**Table 1:** Participant Characteristics in the Shanghai Men's Health Study, 2002–2017

Variables	No BF	OBF	Non‐OBF	*p1* Value^a^	*p2* Value^b^
No. of participants (%)	58,258 (95.4%)	702 (1.2%)	2065 (3.4%)		
Age (years)					
40–50	21,706 (37.3)	210 (29.9)	862 (41.7)		
50–59	18,179 (31.2)	166 (23.7)	693 (33.6)	<0.001	<0.001
60–69	11,607 (19.9)	180 (25.6)	337 (16.3)		
≥70	6766 (11.6)	146 (20.8)	173 (8.4)		
Average age (years), mean ± SD	55.4 ± 9.7	58.5 ± 10.7	54.0 ± 9.1	<0.001	<0.001
Income					
Low	7300 (12.5)	85 (12.1)	280 (13.6)		
Middle	45,311 (77.8)	546 (77.8)	1605 (77.7)	1.000	0.323
High	5647 (9.7)	71 (10.1)	180 (8.7)		
Education					
<High school	23,453 (40.3)	311 (44.3)	844 (40.9)		
High school	20,877 (35.8)	235 (33.5)	774 (37.5)	0.189	0.101
>High school	13,928 (23.9)	156 (22.2)	447 (21.7)		
Smoking status					
Never	17,731 (30.4)	232 (33.0)	559 (27.1)	0.270	0.002
Ever	40,527 (69.6)	470 (67.0)	1506 (72.9)		
Alcohol consumption					
No	38,672 (66.4)	456 (65.0)	1309 (63.4)	0.855	0.010
Yes	19,586 (33.6)	246 (35.0)	756 (36.6)		
BMI (mean ± SD)	23.7 ± 3.1	23.3 ± 3.0	23.7 ± 3.1	0.001	0.357
Regular exercise					
No	37,495 (64.4)	412 (58.7)	1413 (68.4)	0.004	<0.001
Yes	20,763 (35.6)	290 (41.3)	652 (31.6)		
Charlson score					
0	42,941 (73.7)	498 (70.9)	1495 (72.4)		
1	11,541 (19.8)	143 (20.4)	471 (22.8)	0.098	<0.001
≥2	3776 (6.5)	61 (8.7)	99 (4.8)		
History of fractures					
No	43,174 (74.1)	422 (60.1)	1275 (61.7)	<0.001	<0.001
Yes	15,084 (25.9)	280 (39.9)	790 (38.3)		
Multivitamin use					
Non‐users	53,877 (92.5)	642 (91.5)	1912 (92.6)	0.611	1.000
Ever‐users	4381 (7.5)	60 (8.5)	153 (7.4)		
Calcium supplement					
Non‐users	53,691 (92.2)	635 (90.5)	1843 (89.3)	0.190	<0.001
Ever‐users	4567 (7.8)	67 (8.5)	222 (10.7)		
Vitamin D supplement					
Non‐users	57,979 (99.5)	699 (99.6)	2053 (99.4)	1.000	1.000
Ever‐users	279 (0.5)	3 (0.4)	12 (0.6)		
Caloric intake (calories/d), mean ± SD	1914 ± 426	1855 ± 421	1935 ± 445	<0.001	0.017
Protein intake (mg/d), mean ± SD	78.5 ± 20.9	75.7 ± 19.9	80.4 ± 21.9	<0.001	<0.001
Fat intake (mg/d), mean ± SD	34.5 ± 13.6	33.6 ± 13.3	35.7 ± 14.5	0.112	<0.001
Diet calcium (mg/d), mean ± SD	598 ± 217	570 ± 199	612 ± 226	0.001	0.007
Diet magnesium (mg/d), mean ± SD	324 ± 83.6	312 ± 78.3	330 ± 87.9	0.001	0.001
Diet vit D (IU/d), mean ± SD	108 ± 59.8	101 ± 54.8	109 ± 59.5	0.007	0.364

BF = bone fractures; OBF = osteoporotic bone fractures; Non‐OBF = non‐osteoporotic bone fractures; BMI = body mass index.

^a^ *p1* for difference between OBF and no BF.

^b^ *p2* for difference between non‐OBF and no BF.

**Fig. 2:**
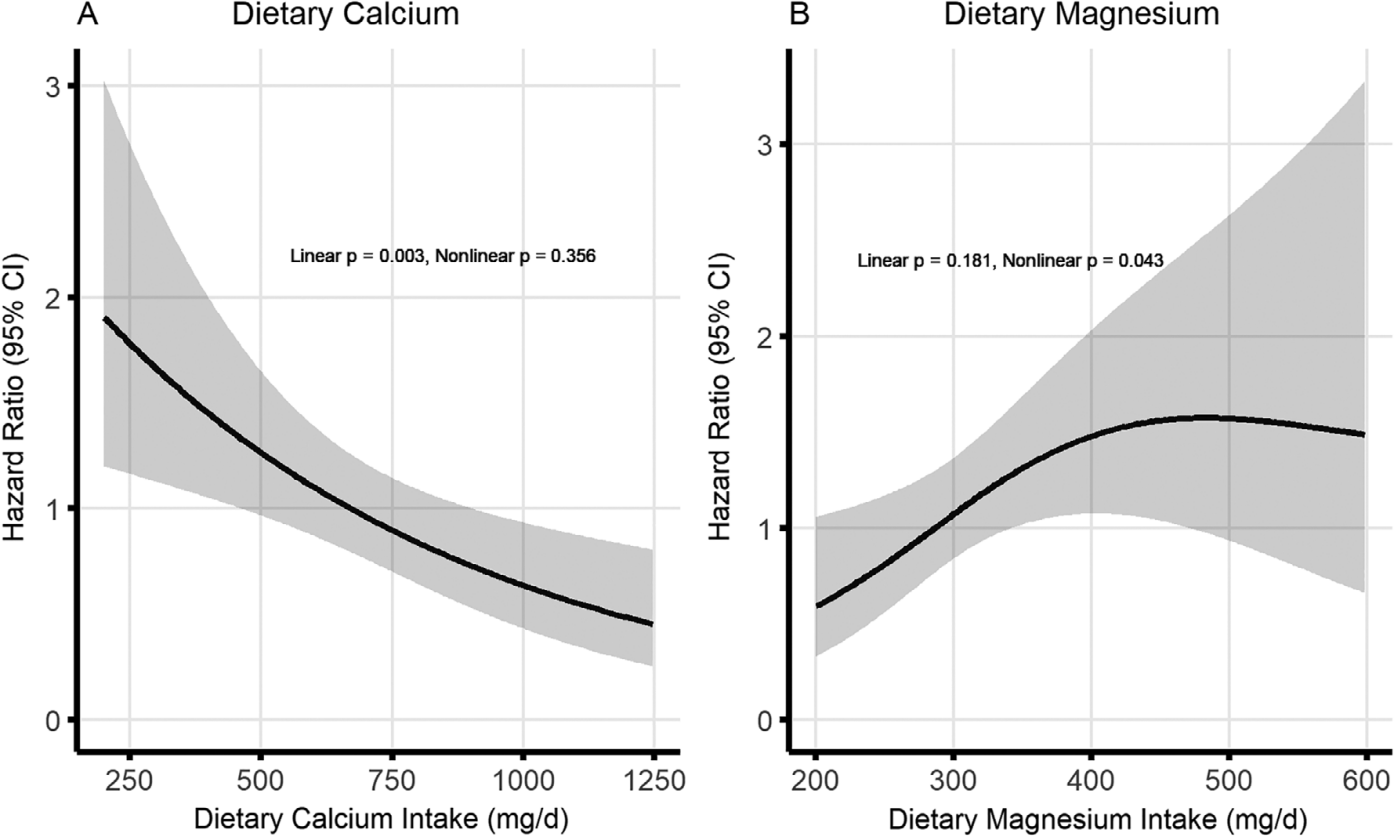
Multivariable adjusted spline curves for relationship between dietary intakes of calcium (*A*) or magnesium (*B*) and time to first osteoporotic fractures. Multivariable adjusted hazard ratios indicated by solid lines and 95% confidence intervals by the shaded area under the curves. The models were adjusted for age at enrollment, educational level, cigarette smoking status, alcohol consumption, regular exercise, body mass index, Charlson score, fracture history at baseline, calcium supplement use, and daily intakes of calories, protein, fat, and vitamin D; dietary calcium and magnesium intakes were adjusted mutually.

Table 2 shows associations between dietary calcium or magnesium intakes and risk of fractures by type. After controlling for sociodemographics, lifestyle‐related factors, medical conditions, use of calcium supplements, and dietary intakes of calories, protein, fat, and vitamin D (model 1), dietary calcium intake was inversely associated with osteoporotic fractures (HRs ranged from 0.84 (95% CI 0.65–1.08) to 0.43 (95% CI 0.24–0.78) from intake levels of 401 mg/d to more than 1000 mg/d, compared with ≤400 mg/d. After additional adjustment for dietary magnesium intake (model 2, full adjustment), the calcium‐osteoporotic fracture association became stronger (corresponding HRs ranged from 0.78 [95% CI 0.60–1.02] to 0.27 [95% CI = 0.13–0.56]). Cubic spline regression analysis revealed that the inverse association between calcium intake and osteoporotic fractures followed a clear linear dose–response pattern (linear *p* = 0.003, Fig. 2*A*). HR associated with each 50 mg increment was 0.95 (95% CI 0.91–0.99; *p* = 0.009) (data not shown in tables). However, no association was observed between calcium intake and non‐osteoporotic fractures, with or without adjustment for magnesium intake. In contrast, higher magnesium intake was associated with increased risk of osteoporotic fractures after adjusting for calcium intake and other covariates (HRs ranged from 2.12 [95% CI = 1.15–3.19] to 2.21 [95% CI = 1.08–4.50]) for intakes of 401 mg/d or higher compared with intake ≤250 mg/d). Fig. 2*B* shows that the association between magnesium intake and osteoporotic fracture risk was non‐linear (non‐linear *p* = 0.043); the risk elevated with increased magnesium intake from 200 mg/d to about 400 mg/d, after which, the risk leveled over. However, magnesium intake was not associated with osteoporotic fractures when calcium intake was not adjusted for. No association was found between dietary magnesium intake and non‐osteoporotic fractures with or without adjustment for dietary calcium intake.

**Table 2:** Associations Between Diet Calcium or Magnesium Intake and Risk of Bone Fractures by Type Among Shanghai Men's Health Study Participants

OBF versus no BF	Events/no BF	Model 1^a^, adj HR (95% CI)	Model 2^b^, adj HR (95% CI)
Calcium intake (mg/d)			
≤400	146/10,024	1.00 (ref)	1.00 (ref)
401–500	126/10,133	0.84 (0.65–1.08)	0.78 (0.60–1.02)
501–600	150/11,468	0.84 (0.65–1.10)	0.75 (0.56–1.02)
601–800	205/17,638	0.72 (0.53–0.98)	0.60 (0.41–0.87)
801–1000	58/6519	0.54 (0.36–0.82)	0.38 (0.23–0.64)
>1000	17/2476	0.43 (0.24–0.78)	0.27 (0.13–0.56)
Magnesium intake (mg/d)			
≤250	136/10,105	1.00 (ref)	1.00 (ref)
251–300	199/14,447	1.09 (0.86–1.39)	1.27 (0.97–1.66)
301–350	171/14,816	0.98 (0.71–1.35)	1.34 (0.91–1.97)
351–400	100/9817	0.91 (0.60–1.40)	1.48 (0.88–2.47)
401–450	59/4951	1.09 (0.66–1.80)	2.12 (1.15–3.91)
>450	37/4122	0.87 (0.50–1.51)	2.21 (1.08–4.50)
Non‐OBF versus no BF			
Calcium intake (mg/d)			
≤400	335/10,024	1.00 (ref)	1.00 (ref)
401–500	334/10,133	0.95 (0.81–1.11)	0.92 (0.78–1.09)
501–600	434/11,468	1.09 (0.93–1.28)	1.05 (0.87–1.27)
601–800	602/17,638	0.98 (0.82–1.18)	0.95 (0.76–1.18)
801–1000	248/6519	1.09 (0.87–1.38)	1.02 (0.76–1.36)
>1000	112/2476	1.24 (0.94–1.64)	1.06 (0.74–1.53)
Magnesium intake (mg/d)			
≤250	313/10,105	1.00 (ref)	1.00 (ref)
251–300	511/14,447	1.08 (0.93–1.26)	1.09 (0.92–1.29)
301–350	543/14,816	1.10 (0.91–1.34)	1.11 (0.88–1.40)
351–400	321/9817	0.99 (0.77–1.28)	1.00 (0.73–1.35)
401–450	192/4951	1.15 (0.86–1.54)	1.14 (0.79–1.63)
>450	185/4122	1.33 (0.98–1.81)	1.27 (0.85–1.91)

BF = bone fractures; OBF = osteoporotic bone fractures; Non‐OBF = non‐osteoporotic bone fractures; HR = hazard ratio; CI = confidence interval.

^a^ Model 1: Dietary calcium or magnesium intake was included in the model, with adjusting for age at enrollment, educational level, cigarette smoking status, alcohol consumption, regular exercise, body mass index, Charlson score, fracture history at baseline, calcium supplement use, daily intakes of calories, protein, fat, and vitamin D.

^b^ Model 2: Adjusting for all covariates included in model 1 and additionally mutually adjusted for dietary calcium and magnesium intakes.

**Fig. 3:**
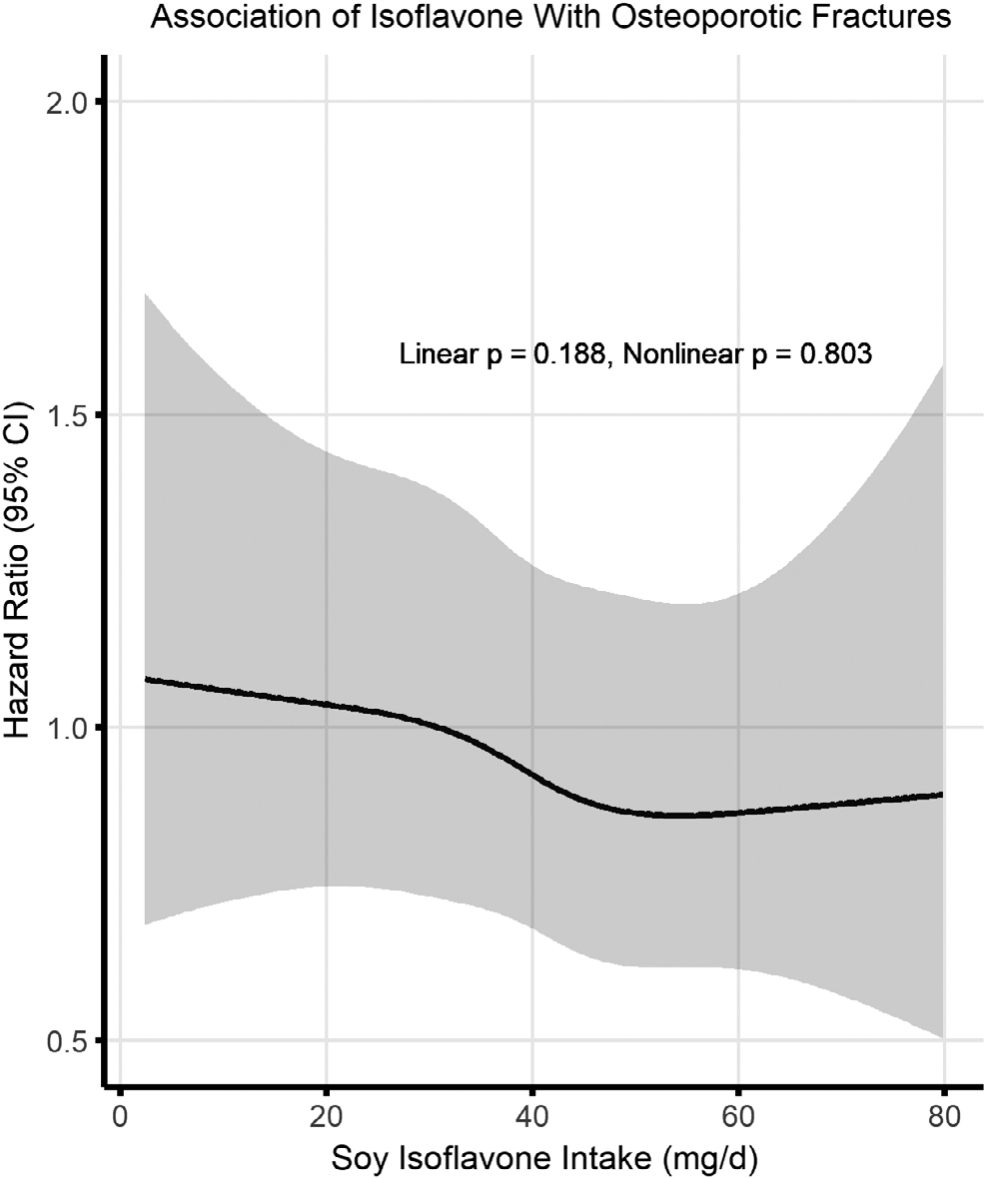
Multivariable adjusted spline curve for relationship between soy isoflavone intake and time to first osteoporotic fractures. Multivariable adjusted hazard ratio indicated by solid line and 95% confidence interval by the shaded area under the curve. The model was adjusted for age at enrollment, educational level, cigarette smoking status, alcohol consumption, regular exercise, body mass index, Charlson score, fracture history at baseline, calcium supplement use, and daily intakes of calories, protein, fat, vitamin D, calcium, and magnesium.

Table 3 shows the association between soy isoflavone intake and risk of fractures. Compared with the lowest quartile (Q1, <21.7 mg/d), the highest quartile (Q4, >45.2 mg/d) of soy isoflavone intake was associated with reduced risk of osteoporotic fractures (HR = 0.70, 95% CI 0.55–0.91 in the analysis without adjustment for calcium and magnesium [model 1]; HR = 0.73, 95% CI 0.56–0.97 in the analysis with additional adjustment for calcium and magnesium [model 2]). Fig. 3 shows that when isoflavone intake was between about 30 mg/d and 50 mg/d, the risk of osteoporotic fractures decreased with increasing intake, after which the risk leveled over. However, overall, there was no significant linear (*p* = 0.188) or non‐linear (*p* = 0.803) dose–response pattern. Similar association patterns were observed between genistein, daidzein, or glycitein intake and osteoporotic fracture risk (HR = 0.72, 95% CI 0.56–0.93 [model 1] and HR = 0.75, 95% CI 0.58–0.99 [model 2] for genistein; HR = 0.75, 95% CI 0.58–0.96 [model 1] and HR = 0.78, 95% CI 0.59–1.02 [model 2] for daidzein; HR = 0.73, 95% CI 0.56–0.94 [model 1] and HR = 0.76, 95% CI 0.57–1.01 [model 2] for glycitein). By contrast, compared with the lowest quartile (Q1, <21.7 mg/d), the higher quartiles (Q3 and Q4) of soy isoflavone intake were associated with a marginally statistically increased risk of non‐osteoporotic fractures (HR = 1.14, 95% CI 0.99–1.30 and HR = 1.14, 95% CI 0.99–1.33 for model 1; HR = 1.15, 95% CI 0.99–1.32 and HR = 1.16, 95% CI 0.98–1.36 for model 2). Similar association patterns were observed between genistein, daidzein, or glycitein intakes and non‐osteoporotic fractures (Table 3).

**Table 3:** Associations of Soy Isoflavone Intake With Bone Fractures by BF Type Among Shanghai Men's Health Study Participants

OBF versus no BF	Events/no BF	Model 1^a^, adj HR (95% CI)	Model 2^b^, Adj HR (95% CI)
Isoflavone intake (mg/d)			
Q1 (<21.7)	197/14,604	1.00 (ref.)	1.00 (ref.)
Q2 (21.7–32.1)	177/14,571	0.89 (0.72–1.09)	0.89 (0.72–1.10)
Q3 (32.2–45.2)	180/14,529	0.89 (0.72–1.12)	0.91 (0.73–1.15)
Q4 (>45.2)	148/14,554	0.70 (0.55–0.91)	0.73 (0.56–0.97)
Genistein intake (mg/d)			
Q1 (<11.8)	198/14,597	1.00 (ref.)	1.00 (ref.)
Q2 (11.8–17.8)	178/14,574	0.89 (0.72–1.09)	0.89 (0.73–1.10)
Q3 (17.9–25.3)	174/14,535	0.86 (0.69–1.07)	0.88 (0.70–1.11)
Q4 (>25.3)	152/14,552	0.72 (0.56–0.93)	0.75 (0.58–0.99)
Daidzein intake (mg/d)			
Q1 (<8.8)	195/14,604	1.00 (ref.)	1.00 (ref.)
Q2 (8.8–13.1)	180/14,573	0.92 (0.74–1.13)	0.93 (0.75–1.14)
Q3 (13.2–18.6)	174/14,534	0.88 (0.71–1.10)	0.90 (0.72–1.13)
Q4 (>18.6)	153/14,547	0.75 (0.58–0.96)	0.78 (0.59–1.02)
Glycitein intake (mg/d)			
Q1 (<1.9)	200/14,597	1.00 (ref.)	1.00 (ref.)
Q2 (1.9–2.6)	176/14,583	0.87 (0.70–1.07)	0.88 (0.71–1.08)
Q3 (2.7–3.7)	171/14,531	0.83 (0.67–1.04)	0.85 (0.67–1.07)
Q4 (>3.7)	155/14,547	0.73 (0.56–0.94)	0.76 (0.57–1.01)
Non‐BF versus no BF			
Isoflavone intake (mg/d)			
Q1 (<21.7)	455/14,604	1.00 (ref.)	1.00 (ref.)
Q2 (21.7–32.1)	508/14,571	1.06 (0.93–1.21)	1.07 (0.94–1.22)
Q3 (32.2–45.2)	548/14,529	1.14 (0.99–1.30)	1.15 (0.99–1.32)
Q4 (>45.2)	554/14,554	1.14 (0.99–1.33)	1.16 (0.98–1.36)
Genistein intake (mg/d)			
Q1 (<11.8)	461/14,597	1.00 (ref.)	1.00 (ref.)
Q2 (11.8–17.8)	504/14,574	1.04 (0.91–1.18)	1.04 (0.91–1.19)
Q3 (17.9–25.3)	548/14,535	1.12 (0.98–1.28)	1.13 (0.98–1.29)
Q4 (>25.3)	552/14,552	1.12 (0.97–1.30)	1.13 (0.96–1.32)
Daidzein intake (mg/d)			
Q1 (<8.8)	458/14,604	1.00 (ref.)	1.00 (ref.)
Q2 (8.8–13.1)	502/14,573	1.05 (0.92–1.19)	1.05 (0.92–1.18)
Q3 (13.2–18.6)	549/14,534	1.13 (0.99–1.29)	1.14 (0.99–1.31)
Q4 (>18.6)	556/14,547	1.15 (0.99–1.33)	1.16 (0.99–1.37)
Glycitein intake (mg/d)			
Q1 (<1.9)	460/14,597	1.00 (ref.)	1.00 (ref.)
Q2 (1.9–2.6)	498/14,583	1.04 (0.91–1.18)	1.04 (0.91–1.20)
Q3 (2.7–3.7)	553/14,531	1.14 (0.99–1.30)	1.15 (0.99–1.32)
Q4 (>3.7)	554/14,547	1.14 (0.98–1.32)	1.16 (0.98–1.37)

BF = bone fractures; OBF = osteoporotic bone fractures; Non‐OBF = non‐osteoporotic bone fractures; HR = hazard ratio; CI = confidence interval.

^a^ Model 1: Adjusting for age at enrollment, educational level, cigarette smoking status, alcohol consumption, regular exercise, body mass index, Charlson score, fracture history at baseline, calcium supplement use, daily intakes of calories, protein, fat, and vitamin D.

^b^ Model 2: Adjusting for all covariates included in model 1 and additionally for dietary calcium and magnesium intakes.

We conducted a sensitivity analysis that excluded 4856 men from the study (approximately 8.0% of all participants) who were calcium supplement users. The results showed the association between osteoporotic fractures and soy isoflavone intake remained largely unchanged (HRs for the highest versus lowest quartile intakes were 0.71, 95% CI 0.54–0.93 for model 1 and 0.74, 95% CI 0.55–0.99 for model 2; data not shown in tables). The associations for dietary calcium and magnesium intakes also remained largely unchanged (data not shown). We also conducted a sensitivity analysis to include 62 men who had both osteoporotic and non‐osteoporotic fractures; among them, 29 men who first experienced osteoporotic fractures were categorized as osteoporotic fractures, and 33 men who first had non‐osteoporotic fractures were categorized as non‐osteoporotic fractures. Results from this sensitivity analysis were similar to those presented in Tables 2 and 3 (data not shown).

## 4 Discussion

In this large‐scale prospective study, with an average age of 55.4 years at study enrollment, we observed that 1.2% of men developed osteoporotic fractures and 3.4% of men had non‐osteoporotic fractures during a median follow‐up time of nearly 10 years. As expected, we found that osteoporotic fractures markedly increased with age and decreased with increase of dietary calcium intake. Such associations were not found for non‐osteoporotic fractures. Dietary magnesium intake, on the other hand, was associated with an increased risk for osteoporotic fractures after adjustment for dietary calcium intake. We found, for the first time, that high levels of total soy isoflavone intake and intakes of major isoflavone components (genistein, daidzein, or glycitein) were associated with a reduced risk of osteoporotic fractures in men, independent of sociodemographic factors and known or suggested risk/protective factors for osteoporotic fractures, including vitamin D, calcium, and magnesium intakes.

It has been well‐established that inadequate calcium contributes to the development of osteoporosis. Dietary calcium intake has been recommended for osteoporosis prevention.^(^ ^10^, ^33^ ^)^ However, a wide range of daily calcium intake has been recommended for individuals based on age and sex, and the recommendation varies by country/organization.^(^ ^10^, ^11^, ^12^ ^)^ Evidence‐based optimal levels for calcium intake for the prevention of osteoporotic fractures, however, are lacking, especially for Asian populations. In general, Asians have lower calcium intake.^(^ ^33^ ^)^ In a previous study of 5307 Chinese adults (men and women) aged 50 to 64 years, the mean calcium intake was reported to be 332.7 mg/d.^(^ ^34^ ^)^ In contrast, the National Health and Nutrition Examination Survey (NHANES), 2003–2006 data, showed that in the United States, males aged 51 to 70 years, on average, had 951 mg/d calcium intake from diet alone and 1092 mg/d from all sources.^(^ ^35^ ^)^ In our study, the mean calcium intake from dietary sources for 61,025 urban Shanghai men, with an average age of 55 years, was about 598 mg/d. We found that dietary calcium intake was inversely associated with incidence of osteoporotic fractures in a dose–response fashion in a range of calcium intake up to more than 1000 mg/d. Our finding supports the NIH's recommendation of 1000 mg/d of dietary calcium intake for osteoporosis prevention. Furthermore, about 75% of participants in our study had dietary calcium intake of less than 720 mg/d, and only 8.0% consumed calcium supplements, suggesting that the majority of men living in Shanghai, one of the most developed cities in China, do not reach an optimal level of calcium intake to prevent osteoporotic fractures.

Previous studies examining the associations of dietary magnesium intake with osteoporosis and risk of fractures are limited and have yielded mixed results. The results from the Women's Health Initiative Observation Study found that a lower magnesium intake was associated with lower bone mineral density (BMD) of the hip and whole body; however, this did not translate to an increased risk of fractures. In the same study, excess magnesium appeared to be detrimental to bone and fracture risk of the forearm and wrist.^(^ ^14^ ^)^ A meta‐analysis showed that high magnesium intake was not associated with increased fracture risk; however, a positive marginally significant correlation was found between magnesium intake and BMD in the total hip as well as in the femoral neck.^(^ ^36^ ^)^ In our study, we found that without adjusting for dietary calcium intake, magnesium intake was not associated with osteoporotic fractures; however, when mutually adjusting for dietary calcium and magnesium intakes in the analyses, magnesium intake was positively associated with osteoporotic fractures. The inverse association between calcium intake and osteoporotic fractures became stronger when adjusting for magnesium intake. Magnesium is a natural calcium antagonist, and the effects of magnesium or calcium on bone health are dependent on the intake amount of calcium or magnesium and vice versa.^(^ ^15^ ^)^ Failure to take these properties into consideration when evaluating the health effects of calcium or magnesium may lead to erroneous conclusions.

Several studies have reported that dietary isoflavones attenuate menopause‐induced osteoporotic bone loss and fractures among women.^(^ ^20^, ^21^, ^22^, ^23^, ^24^, ^25^, ^37^ ^)^ For example, soy isoflavone intake was associated with a reduced risk of incident fractures among healthy postmenopausal women shortly after onset of menopause^(^ ^23^ ^)^ and in pre‐/perimenopausal breast cancer survivors.^(^ ^24^ ^)^ A review of randomized controlled trials suggested that soy isoflavone consumption during menopausal transition may prevent a reduction in bone mineral density and promote bone health.^(^ ^25^ ^)^ This beneficial effect was mainly attributed to isoflavone's estrogenic/antiestrogenic effects in women. However, a study on the effect of soy isoflavone intake on osteoporotic fractures in men is lacking. Our study provides the first evidence that soy isoflavone intake is associated with reduced osteoporotic fractures in men. In vitro experiments and in vivo animal studies have shown that, besides potent estrogenic activity, isoflavones also possess a number of biologic effects that may maintain or improve bone health, such as antioxidant and immune‐modulating effects, anti‐inflammatory activity, anti‐parathyroid hormone activity, and the ability to inhibit bone resorption and stimulate bone formation.^(^ ^6^, ^38^, ^39^, ^40^ ^)^ These biological mechanisms may explain the association between soy isoflavone intake and reduced risk of osteoporotic fractures in men observed in our study.

The strengths of this study include the prospective study design, large sample size, high response rates, repeated dietary assessments using a validated FFQ, and parallel analyses of non‐osteoporotic fractures. However, our study also has several limitations. First, our information on fracture incidences and exposures was self‐reported. No information was available on BMD and history of osteoporotic fractures. Thus, misclassification on outcome assessment is possible. In this study, we defined osteoporotic fracture as low‐trauma bone fractures (eg, due to falls by sliding/fall from standing height) and occurring in anatomic sites commonly associated with osteoporosis, which takes into consideration both cause and anatomic sites to minimize the outcome misclassification bias. We found that participants within osteoporotic fracture or non‐osteoporotic fracture groups showed significant differences in age‐specific incidence, as well as in several other socioeconomic and lifestyle factors, in addition to the different association patterns with calcium, magnesium, and isoflavones. These data provide indirect support to the validity of our assessment of osteoporotic fractures, as well as our study findings. Second, since our study was not originally designed for investigating osteoporotic fractures, information on a number of potential risk factors for osteoporosis and osteoporotic fractures, such as a family history of osteoporosis and hyperthyroidism or hyperparathyroidism, was not collected in the study. Thus, residual confounding may remain.

Our study shows that dietary calcium intake was inversely associated with incidence of osteoporotic fractures following a linear dose–response pattern, whereas high dietary magnesium intake was related to increased risk after controlling for dietary calcium intake. We also provide the first evidence that a high level of soy isoflavone intake was associated with a reduced risk of osteoporotic fractures in men, independently of known risk/protective factors, including dietary calcium and magnesium intakes. While these findings, if confirmed, have a direct impact on development strategies to prevent against osteoporosis and osteoporotic fractures among Chinese men, they also provide novel information regarding the possible role of dietary intake on male bone health in general.

## Disclosures

All authors state that they have no conflicts of interest.

## Author Contributions

**Yong Cui:** formal analysis; investigation; methodology; writing ‐ original draft; writing ‐ review and editing. **Hui Cai:** formal analysis; methodology; writing ‐ review and editing. **Wei Zheng:** conceptualization; formal analysis; funding acquisition; writing ‐ review and editing. **Xiao‐Ou Shu:** conceptualization; formal analysis; funding acquisition; methodology; supervision; writing ‐ original draft; writing ‐ review and editing.

5

### Peer Review

The peer review history for this article is available at https://publons.com/publon/10.1002/jbm4.10563.
